# MicroRNA-145-Mediated KDM6A Downregulation Enhances Neural Repair after Spinal Cord Injury *via* the NOTCH2/Abcb1a Axis

**DOI:** 10.1155/2021/2580619

**Published:** 2021-05-25

**Authors:** Changzhao Gao, Fei Yin, Ran Li, Qing Ruan, Chunyang Meng, Kunchi Zhao, Qingsan Zhu

**Affiliations:** Department of Spine Surgery, China-Japan Union Hospital of Jilin University, Changchun 130033, China

## Abstract

Spinal cord injury (SCI) causes a significant physical, emotional, social, and economic burden to millions of people. MicroRNAs are known players in the regulatory circuitry of the neural repair in SCI. However, most microRNAs remain uncharacterized. Here, we demonstrate the neuroprotection of microRNA-145 (miR-145) after SCI *in vivo* and *in vitro*. In silico analysis predicted the target gene KDM6A of miR-145. The rat SCI model was developed by weight drop, and lipopolysaccharide- (LPS-) induced PC12 cell inflammatory injury model was also established. We manipulated the expression of miR-145 and/or KDM6A both *in vivo* and *in vitro* to explain their roles in rat neurological functional recovery as well as PC12 cell activities and inflammation. Furthermore, we delineated the mechanistic involvement of NOTCH2 and Abcb1a in the neuroprotection of miR-145. According to the results, miR-145 was poorly expressed and KDM6A was highly expressed in the spinal cord tissue of the SCI rat model and LPS-induced PC12 cells. Overexpression of miR-145 protects PC12 cells from LPS-induced cell damage and expedites neurological functional recovery of SCI in rats. miR-145 was validated to target and downregulate the demethylase KDM6A expression, thus abrogating the expression of Abcb1a by promoting the methylation of NOTCH2. Additionally, *in vivo* findings verified that miR-145 expedites neuroprotection after SCI by regulating the KDM6A/NOTCH2/Abcb1a axis. Taken together, miR-145 confers neuroprotective effects and enhances neural repair after SCI through the KDM6A-mediated NOTCH2/Abcb1a axis.

## 1. Introduction

Spinal cord injury (SCI) is recognized as a severe neurological disorder, which results in sensory, motor, and autonomic deficits [1, 2]. The permanent neurological deficits have been attributed to SCI-induced dysfunction of neural connectivity [3]. Approximately 300,000 people suffer from SCI in the United States, and no effective therapies have been developed to reverse the neurological impairments, leading to an urgent call for treatment options to rescue damaged neurons or to enhance neuroplasticity and functional recovery [4]. SCI is accompanied by a wide array of fatal complications during the acute and long-term phases [5]. The serious and irreversible deficits derived from SCI induce inflammatory and pathological processes [6]. It is therefore critical to study novel molecular mechanisms in order to induce a neuroprotective condition to prevent the exacerbation of the secondary neurological function injury [7].

In recent years, we have witnessed a plethora of studies proposing the ability of microRNAs (miRNAs) in restoring or deteriorating neuroplasticity following SCI [8, 9]. SCI can result in alterations of serum miRNAs, and these aberrantly expressed miRNAs and related signaling may illuminate the pathophysiological mechanism of SCI [10]. For example, a prior study suggested the protective effect of miR-127-5p on motor functional recovery following SCI, which was achieved by binding to a target gene MAPK1 [11]. Of note, microRNA-145 (miR-145) has been indicated to prevent LPS-induced injury in PC12 cells through counteracting cell apoptosis and inflammation [12]. In this study, the targeting relationship between miR-145 and lysine demethylase 6A (KDM6A) was predicted by an initial bioinformatics. KDM6A (also known as UTX), as a histone demethylase, has been identified to express highly following SCI, and the ablation of KDM6A was shown to restore neurological function after SCI [13]. Furthermore, it is noted that KDM6A can augment NOTCH receptor 2 (NOTCH2) expression through its demethylase function [14]. Evidence has been provided demonstrating that inactivation of the NOTCH signaling pathway could accelerate the proliferation of neural stem cells, thereby leading to enhanced repair post-SCI [15]. In addition, ATP binding cassette subfamily B member 1A (Abcb1a), also known as P-gp, is a drug efflux transporter of the blood-spinal cord barrier, and its inhibition results in anti-inflammatory effects and reduced drug resistance following SCI [16]. Therefore, this study is aimed at delineating new mechanisms by which the miR-145-mediated KDM6A/NOTCH2/Abcb1a axis exerts its neuroprotective effects post-SCI.

## 2. Materials and Methods

### 2.1. Ethics Statement

The animal experimental processes were approved by the Ethnic Committee of Animal Experiment in School of Public Health, Jilin University, and in accordance with the Animals in Research: Reporting In Vivo Experiments (ARRIVE) guidelines (https://arriveguidelines.org/).

### 2.2. Establishment of Nontraumatic SCI In Vitro Cell Model

PC12 cells (ATCC, Manassas, VA, USA) were routinely placed in RPMI-1640 medium. RPMI-1640 medium containing 10% fetal bovine serum (both from Gibco, Grand Island, NY) was adopted to make a complete growth medium. These cells were maintained in an environment of 5% CO_2_. To stimulate inflammatory damage, cells were treated with 5 *μ*g/mL lipopolysaccharide (LPS) (Sigma-Aldrich, St. Louis, MO) for 12 h [17].

### 2.3. Establishment of Rat SCI Model

Forty adult male SD rats (250-300 g; 6-8 weeks) were included in this study, with an average weight of 271.13 ± 9.73 g and an average age of 7.05 ± 0.80 weeks, were purchased from the Center of Laboratory Animals, Jilin University, China (license No. SCXK(Ji)2008-0005). The rats were bred and maintained in a 12 : 12-h light-dark cycle, with free access to food and water. The room temperature was 25°C, and the relative humidity was 40-60%. All surgeries were performed under anesthesia, and every effort was made to reduce pain. Forty rats were evenly classified into 5 groups (*n* = 8). All animals, with free access to water, were fasted for 12 h before the operation. One group of rats were sham operated with laminectomy, but the T_10_ spinal cord was continuously separated, and then muscles, fascia, and skin were sutured in order. The rest were subjected to SCI modeling, and they underwent T_10_ spinal cord dissection after laminectomy.

The SCI model was developed using the modified Allen method [18]. The rats were anesthetized intraperitoneally with 0.3% sodium pentobarbital (30 mg/kg). After anesthesia, the rats were fixed in a prone position. A longitudinal skin incision of about 4 cm in length was made with position T_10_ as the center to sequentially cut through each layer of subcutaneous tissue to expose the spinous process of T_10_. The spinous process of the T_10_ spine and the lamina were removed with a microbone forceps, and the T_10_ spinal cord was exposed. A thin circular plastic spacer with a diameter of about 3.5 mm on the striking surface was first placed on the surface of the T_10_ spinal cord. Then, a 10 g hammer was employed to fall freely from a height of 30 mm, which hit the dural sac, causing an impact injury to the T_10_ spinal cord. After the impact, the injured spinal cord was washed with sterile warm saline, and then muscles, fascia, and skin were sutured in order. Signs of successful SCI model building were described as follows. When the Allen device hit the spinal cord, the rat body was shaking, its lower limbs quickly retracted and bounced, its tail lifted up and fell down quickly, and it hit the local spinal cord surface to cause rapid blood stasis. After the application of antibiotics, intramuscular injection of penicillin was 30,000 U/time for 3 consecutive days, twice daily. After acute SCI, the rats will temporarily lose their voluntary urination function. The bladder of lower abdomen was pressed to perform artificial urination, 3 times a day, until the rats' voluntary urination function was restored. Basso, Beattie, and Bresnahan (BBB) scales were employed to evaluate the functional recovery of the motor capacity of rats within 6 weeks after SCI.

### 2.4. Adenovirus-Mediated Injection

ADV4(CMV/IRES-GFP) adenoviral vectors overexpressing miR-145 or carrying negative control (NC) plasmids were purchased from Shanghai GenePharma (Shanghai, China). Then, SCI rats were randomly divided into 4 groups (*n* = 8) and, respectively, subjected to SCI, SCI+miR-145+overexpression- (oe-) NC, SCI+miR-NC+oe-NC, and SCI+miR-145+oe-KDM6A. Adenovirus (10^8^ pfu/mL) was injected into the injured spinal cord of a SCI rat at a distance of 0.5 cm from the caudal and anterior sides of the lesion. The adenovirus-mediated injection was firstly performed at the first day after the SCI operation and then once a day for 10 days.

### 2.5. Neurological Function Assessment

Neuronal function recovery was assessed 3 and 7 days after SCI, respectively. Rats were examined by two observers who were blind to the group treatment. Hindlimb motor function was evaluated according to the BBB exercise capacity rating scale [19]. Scoring ranges from 0 (complete paralysis) to 21 (normal exercise). The BBB score was employed to classify the combination of hindlimb motion and joint motion, weight support, forelimb/hindlimb coordination, trunk position and stability, stepping, paw position, toe clearance, and tail position. These combinations represented a continuous recovery phase for rats after SCI.

### 2.6. Tissue Sampling and Section Preparation

Animals in each group were anesthetized at 4 and 7 days after SCI, and 200 mL of 0.1 mol/L phosphate-buffered saline (PBS; pH 7.4) was administered. Then, 400 mL of 4% paraformaldehyde in PBS (pH 7.4) was added. The entire spinal cord was dissected, and the snout/caudal position was marked. A 3 cm piece of tissue was removed from the spinal cord at the center of the injury. The spinal cord tissue was immersed in 0.1 mol/L phosphate buffer solution of 4% paraformaldehyde and stored at 4°C for 1 week. Paraffin-embedded specimens were cut into 5 *μ*m thick sections. To assess histopathological changes, sections were further stained with hematoxylin and eosin (HE).

### 2.7. Nissl Staining

These slides were dewaxed, rehydrated, stained in 0.5% toluidine blue solution (50°C) for 10 min, and rinsed quickly in distilled water three times. The slides were dehydrated and cleared twice with xylene and fixed with a neutral balsam. Image processing software (Media Cybernetics, Rockville, MD, USA) was employed to analyze neuron images captured under a light microscope.

### 2.8. TUNEL Staining

The spinal cord was fixed, embedded and cut, fixed with 4% paraformaldehyde for 15 min, and permeabilized in 0.1% Triton-X 100 for 3 min. Then, spinal cord cells were stained with TUNEL staining kit and then counterstained with DAPI for 10 min. The images were observed with a confocal microscope. Then, the percentage of cell apoptosis was calculated to evaluate the degree of apoptosis.

### 2.9. ELISA

The cells were stimulated by LPS, and the culture supernatant was collected. The supernatant was determined for the levels of IL-1*β* (ab217608, Abcam, UK), IL-6 (ab178013, Abcam, UK), IL-8 (ab46032, Abcam, UK), TNF-*α* (ab181421, Abcam, UK), and other proinflammatory cytokines using corresponding ELISA detection kits. The index was read immediately at 450 nm, and the cytokine concentration was calculated according to the standard curve.

### 2.10. RNA Extraction and qRT-PCR

Real-time PCR was employed to measure miRNA and mRNA levels in the spinal cord tissue and PC12 cells. Total RNA was extracted using RNeasy Mini Kit (Qiagen, Valencia, CA, USA), and mRNA was determined by reverse transcription using a reverse transcription kit (RR047A, Takara, Japan) to obtain cDNA. For the detection of microRNA, miRNA First-Strand cDNA Synthesis (Tailing Reaction) kit (B532451-0020, Sangon Biotech Co., Ltd., Shanghai, China) was employed for reverse transcription to obtain cDNA. Samples were loaded using SYBR® Premix Ex TaqTM II (Perfect Real Time) kit (DRR081, Takara, Japan), and the samples were subjected to qRT-PCR reaction in a real-time quantitative PCR instrument (ABI 7500, ABI, Foster City, CA, USA). The expression levels were quantified using a 7500-type real-time PCR instrument from ABI corporation. The primer sequences were shown in Table 1. The Ct value was measured during the exponential amplification phase. The relative expression level of the gene to be tested was the relative change of the 2^-*ΔΔ*Ct^.

### 2.11. Western Blots

Total protein in tissues and cells was extracted with RIPA lysis buffer (R0010, Solarbio) containing PMSF. The cells were incubated for 30 min on ice, centrifuged at 12000 r/min, and 4°C for 10 min, and the supernatant was taken. The BCA kit (23225, Pierce) was employed to determine protein concentration of each sample. A 10% SDS-PAGE (P0012A, Beyotime Institute of Biotechnology, Shanghai, China) was adopted to dissolve proteins, which were transferred to a PVDF membrane (ISEQ00010, Millipore, Billerica, MA, USA) by wet transfer. The PVDF membrane was blocked with TBST buffer containing 5% skimmed milk powder for 2 h. The blots were probed with the primary antibody to rabbit KDM6A (1 : 1000, ab36938, Abcam, UK), NOTCH2 (1 : 1000, Ak098372, Abcam, UK), Abcb1a (1 : 1000, ab3373, Abcam, UK), IL-1*β* (1 : 1000, S328, NIBSC, UK), IL-6 (1 : 1000, Tyr705, Abcam, UK), IL-8 (1 : 1000, S333, Abcam, UK), TNF-*α* (1 : 1000, C432, PA, USA), Bcl-2 (1 : 1000, ab32124, Abcam, UK), Bax (1 : 1000, 6A7, Abcam, UK), caspase-3 (1 : 1000, H-277, Abcam, UK), and *β*-actin (1 : 1000, AC15, Abcam, UK). HRP-labeled goat anti-rabbit IgG antibody (Beijing Zhongshan Biotechnology Co., Ltd., Beijing, China, 1 : 5000) was then incubated with cells. The blots were developed with the ECL and quantified with Quantity One v4.6.2 software.

### 2.12. CCK-8 Assay

Cells were seeded in 96-well plates at 3 × 10^3^ cells/well and treated with LPS (10 ng/mL) for 12 h. Then, 10 *μ*L of CCK-8 working solution was added to each well, and culture was continued at 37°C for 4 h. The blank well was adopted for zero calibration. The microplate reader was employed to measure the absorbance of each well at 570 nm, and the results were recorded. The quantitative detection was performed daily for 7 consecutive days.

### 2.13. Flow Cytometry

Forty-eight hours after cell transfection, 0.25% trypsin (without EDTA) was employed to digest the cells, which were centrifuged to discard the supernatant. The cells were washed 3 times with cold PBS, and the supernatant was discarded by centrifugation. According to the instructions of Annexin-V-FITC Cell Apoptosis Detection Kit (556547, Shanghai Shuojia Biotechnology Co., Ltd., China), Annexin-V-FITC, PI, HEPES buffer solution was formulated into Annexin-V-FITC/PI staining solution. Cells were resuspended with 1 × 10^6^ cells per 100 *μ*L of the staining solution, which were incubated with 15 mL of HEPES buffer solution at room temperature for 15 min. FITC and PI fluorescence were determined by 515 nm and 620 nm bandpass filters excited at 488 nm wavelength, and apoptosis was evaluated.

### 2.14. Dual Luciferase Reporter Assay

The biological prediction website and luciferase reporter assay were employed to verify the targeting relationship between miR-145 and KDM6A. The binding site analysis of miR-145 and KDM6A was performed, and the fragment sequences containing the action site were obtained. The 3′UTR region of KDM6A and the sequence of site-directed mutagenesis with miR-145 binding site were cloned into the target sequence of the psiCheck2 plasmid downstream of the luciferase reporter gene, respectively, which were named KDM6A-WT and KDM6A-MUT, respectively. NC mimic and miR-145 mimic were cotransfected with the luciferase reporter vector, and luciferase activity was measured using a luciferase assay kit (Promega, Madison, WI, USA). After 48 h of incubation, the cells were lysed in 1× Passive lysis. The Dual Luciferase Reporter Assay System (Promega, USA) was employed to measure firefly luciferase activity with Renilla luciferase activity as the internal reference.

### 2.15. ChIP Assay

Cells (1 × 10^7^) were cross-linked with 1% formaldehyde and immunoprecipitated with 1-5 *μ*g KDM6A or H3K27me3 antibody at 4°C overnight. The DNA was then purified, and the NOTCH2 promoter-specific primers were employed to analyze the DNA-enriched template using qRT-PCR.

### 2.16. HE Staining

T9-T12 spinal cord segments centering around and surrounding the injured site were collected and fixed overnight at 4°C in 4% paraformaldehyde. Then, the spinal cord was embedded in paraffin and sectioned. The longitudinal section of the spinal cord was subjected to HE staining and observed with an optical microscope (Olympus B61, Tokyo, Japan).

### 2.17. Statistical Analysis

Measurement data are shown as mean ± standard deviation and analyzed by SPSS 21.0 software (IBM, Armonk, NY, USA), with *p* < 0.05 as a level of statistical significance. The data comparison between two groups was performed using independent sample *t*-test. The comparison among multiple groups was performed by one-way analysis of variance (ANOVA) with Tukey's post hoc test. Statistical analysis in relation to time-based measurements within each group was realized using repeated measures ANOVA, followed by Bonferroni's post hoc test.

## 3. Results

### 3.1. miR-145 Expedites Repair of SCI in Rats

**Figure 1:**
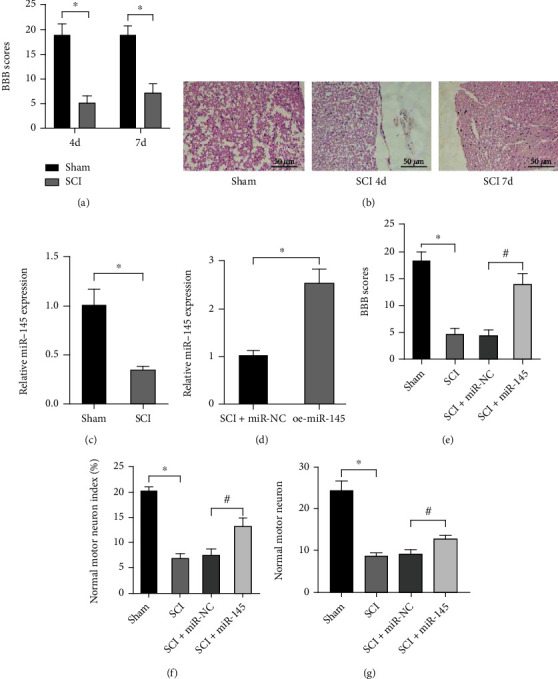
miR-145 expedites repair of SCI in rats. (a) Quantitative analysis for BBB score of neurological function after SCI. (b) HE staining of spinal cord tissue at the injury site (×200). (c) Relative expression level of miR-145 in the rat spinal cord tissue after qRT-PCR detection. (d) miR-145-shuttled adenovirus was injected to rats on the 4th day from the SCI modeling, and the spinal cord tissues were collected 4 weeks later for qRT-PCR determination of miR-145 expression. (e) BBB score to evaluate the neurological function of rats in response miR-145 overexpression. (f) HE staining to assess the normal motor neuron index in all motor neurons (normal+abnormal neurons). (g) Quantitative analysis for number of normal motor neurons in the spinal cord by Nissl staining. Unpaired *t*-test was employed for data comparison between two groups. Data comparison among multiple groups was performed using one-way ANOVA with Tukey's post hoc test. ^∗^*p* < 0.05 versus sham-operated rats, ^#^*p* < 0.05 versus SCI rats with miR-145 NC (*F* = 4.25, df = 2, *p* = 0.023), *n* = 8.

To illuminate the potential role of miR-145 in SCI, we established a SCI rat model and identified its successful establishment through neurological function assessment of BBB scoring and spinal cord pathological changes (Figures 1(a) and 1(b)). Then, we employed qRT-PCR to measure miR-145 expression in the spinal cord tissues (1 cm around the injury site) of rats and identified downregulated expression of miR-145 in the spinal cord tissues of SCI rats (Figure 1(c)).

We then employed adenoviruses to stably overexpress miR-145 and injected the adenovirus overexpressing miR-145 into rats four days after modeling. Four weeks after injection, we explored the neuroprotective effect of miR-145 on SCI rat models. The qRT-PCR results showed that miR-145 levels were higher in the presence of oe-miR-145 in the SCI rats than those with miR-145 NC (*p* < 0.05) (Figure 1(d)). Seven days after the operation, neurological function was assessed using the BBB score. No motor and sensitive dysfunction was observed in the sham-operated rats. The BBB score in the untreated SCI rats was the same as that in the SCI rats with miR-145 NC, whereas the BBB score was elevated after operation in SCI rats with miR-145 overexpression (Figure 1(e)). Next, we employed HE staining and Nissl staining to measure histopathological changes. HE staining (Figure 1(f)) showed that the sections for sham-operated rats displayed that neurons were abundant. The untreated SCI rats and SCI rats with miR-145 NC showed a small number of neurons. A large number of neurons was identified in the SCI rats with miR-145 overexpression, and the cell structure of this group was better than that in the SCI rats with miR-145 NC (Figure 1(f)). Nissl staining (Figure 1(g)) showed that the number of neurons was elevated in SCI rats in response to miR-145; abnormal neuron morphology was observed in untreated SCI rats and SCI rats treated with miR-145 NC, whereas such abnormal morphology could be alleviated by miR-145 overexpression. Together, these data suggest the involvement of miR-145 in nerve repair after SCI.

### 3.2. Overexpression of miR-145 Protects PC12 Cells from LPS-Induced Cell Damage

**Figure 2:**
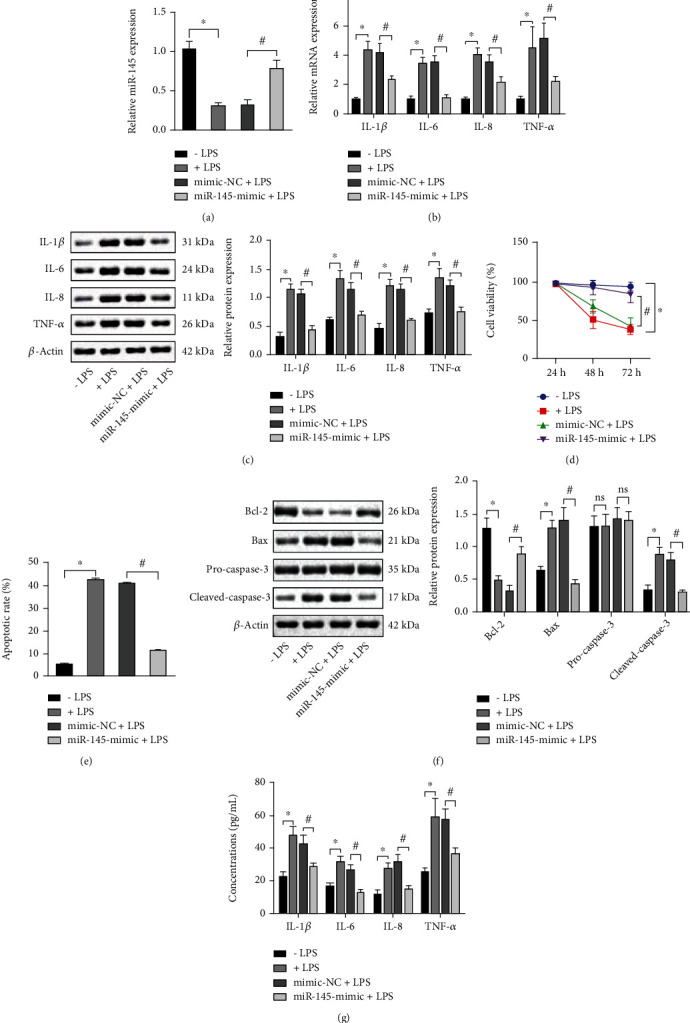
Overexpression of miR-145 protects PC12 cells from LPS-induced cell damage. (a) qRT-PCR detection of miR-145 expression in PC12 cells after transfection with miR-145 mimic. (b) qRT-PCR detection of mRNA levels of proinflammatory cytokines (IL-1*β*, IL-6, IL-8, and TNF-*α*) in response to LPS treatment and miR-145 mimic. (c) Western blot assay of protein levels of proinflammatory cytokines (IL-1*β*, IL-6, IL-8, and TNF-*α*) in response to LPS treatment and miR-145 mimic. (d) CCK-8 assay to measure cell viability in response to LPS treatment and miR-145 mimic. (e) Flow cytometry to measure cell apoptosis in response to LPS treatment and miR-145 mimic. (f) Western blot assay to measure protein levels of apoptosis-related factors in response to LPS treatment and miR-145 mimic. (g) ELISA to measure proinflammatory cytokine release concentration in a cell culture supernatant in response to LPS treatment and miR-145 mimic. Data were expressed as mean ± standard deviation. Unpaired *t*-test was employed for data comparison between two groups. Data comparison among multiple groups was performed using one-way ANOVA with Tukey's post hoc test. ^∗^*p* < 0.05 versus the -LPS group, ^#^*p* < 0.05 versus the mimic-NC+LPS group. Cell experiments were repeated three times.

To explore the molecular regulatory network of miR-145 in neural repair after spinal cord injury, an *in vitro* cell model of nontraumatic SCI was induced PC12 cells by LPS. After miR-145 mimics were transfected into PC12 cells, miR-145 expression was determined in LPS-treated and untreated PC12 cells, respectively. As shown in Figure 2(a), LPS treatment downregulated miR-145 expression, while miR-145 mimics elevated miR-145 expression. It was found that LPS treatment elevated the concentration of proinflammatory cytokines (IL-1*β*, IL-6, IL-8, and TNF-*α*) and overexpressed miR-145 diminished them (Figures 2(b) and 2(c)). The CCK-8 assay displayed that LPS treatment diminished the viability of PC12 cells, while overexpressing miR-145 could protect the LPS-induced cells from damage by LPS treatment by increasing cell viability (Figure 2(d)). Flow cytometric data revealed that LPS treatment elevated the percentage of apoptotic PC12 cells, while overexpression of miR-145 lowered the cell apoptosis (Figure 2(e)). Consistent results were identified by Western blot analysis, where LPS treatment downregulated Bcl-2 protein expression and enhanced Bax and cleaved-caspase-3 expression, while overexpression of miR-145 could elevate Bcl-2 protein expression and restrict Bax and cleaved-caspase-3 expression (Figure 2(f)). ELISA detection (Figure 2(g)) of concentrations of proinflammatory cytokines (IL-1*β*, IL-6, IL-8, and TNF-*α*) in a cell supernatant suggested that miR-145 can reduce the secretion of proinflammatory cytokines. Taken together, miR-145 overexpression was observed to elevate cell viability and repress apoptosis and inflammation, thereby protecting PC12 cells from LPS-induced cell damage.

### 3.3. miR-145 Targets KDM6A

**Figure 3:**
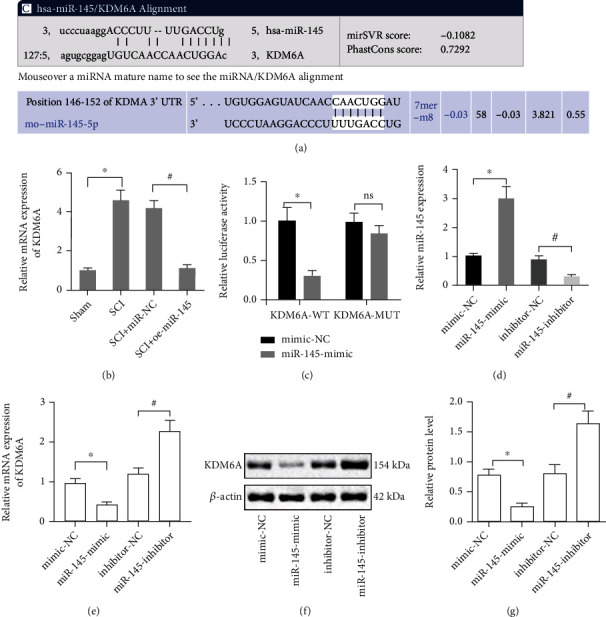
miR-145 targets KDM6A. (a) Bioinformatic website to predict the binding sites between miR-145 and KDM6A. (b) qRT-PCR detection of the relative expression of KDM6A mRNA in the spinal cord tissue of rats (*n* = 8) in response to miR-145 mimic. (c) Dual luciferase reporter assay to validate binding of miR-145 to KDM6A. (d) qRT-PCR to measure miR-145 expression in response to miR-145 mimic and miR-145 inhibitor. (e) qRT-PCR to measure KDM6A mRNA expression in response to miR-145 mimic and miR-145 inhibitor. (f, g) Western blots (f) and statistics (g) of KDM6A protein expression in response to miR-145 mimic and miR-145 inhibitor. Data were expressed as mean ± standard deviation. Independent sample *t*-test was employed for data comparison between two groups. ^∗^*p* < 0.05 versus the mimic-NC group, ^#^*p* < 0.05 versus the inhibitor-NC group (*F* = 5.25, df = 5, *p* = 0.013). Cell experiments were repeated three times.

To determine the regulatory network of miR-145 in the repair of SCI, we searched the TargetScan website (http://www.microrna.org/microrna/home.do) and the microRNA website (http://www.targetscan.org/vert_71/) and predicted the target gene KDM6A of miR-145 in humans and rats (Figure 3(a)). We further sought to investigate whether the role of miR-145 in the repair of SCI was achieved by regulating KDM6A. The data suggested that the expression of KDM6A in the rat models of SCI was higher than that in the sham-operated rats, and the expression of KDM6A in the spinal cord of model rats overexpressing miR-145 was lower than that in the SCI rats with miR-145 NC (Figure 3(b)). Furthermore, the target regulatory relationship was verified using the dual luciferase reporter assay. Relative to the mimic-NC group, the luciferase activity was abrogated in KDM6A 3′UTR WT of miR-145 mimic-transfected cells (*p* < 0.05), while the KDM6A 3′UTR MUT had no significant difference in luciferase activity (*p* > 0.05), indicating that miR-145 can target the KDM6A gene (Figure 3(c)). According to qRT-PCR and Western blot results, the level of miR-145 was increased in the presence of miR-145 mimic (Figure 3(d)), and the levels of KDM6A mRNA (Figure 3(e)) and protein (Figures 3(f) and 3(g)) were decreased. Moreover, miR-145 inhibitor led to the downregulated level of miR-145 (Figure 3(d)), and the expression of KDM6A mRNA (Figure 3(e)) and protein (Figures 3(f) and 3(g)) was upregulated (*p* < 0.05).

### 3.4. miR-145 Protects PC12 Cells from LPS-Induced Cell Damage through KDM6A

**Figure 4:**
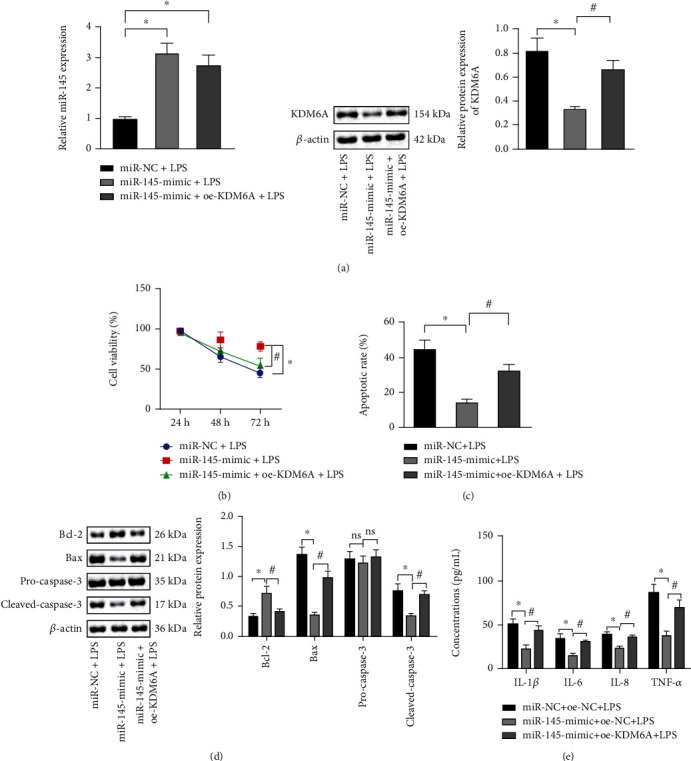
miR-145 protects PC12 cells from LPS-induced cell damage through KDM6A. (a) qRT-PCR to measure miR-145 expression (top), and Western blot assay to measure KDM6A protein expression (bottom) in response to miR-145 mimic and KDM6A overexpression. (b) CCK-8 assay to measure viability of LPS-treated PC12 cells in response to miR-145 mimic and KDM6A overexpression. (c) Flow cytometry to assess apoptosis of LPS-treated PC12 cells in response to miR-145 mimic and KDM6A overexpression. (d) Western blot assay of protein levels of apoptosis-related factors in response to miR-145 mimic and KDM6A overexpression. (e) ELISA detection of proinflammatory cytokine release in cell culture fluid in response to miR-145 mimic and KDM6A overexpression. Data were expressed as mean ± standard deviation. Data comparison among multiple groups was performed using one-way ANOVA with Tukey's post hoc test. Statistical analysis in relation to time-based measurements within each group was realized using repeated measures ANOVA, followed by a Bonferroni's post hoc test. ^∗^*p* < 0.05 versus the LPS+mimic-NC+oe-NC group, ^#^*p* < 0.05 versus the miR-145-mimic+oe-NC group (*F* = 5.65, df = 3, *p* = 0.034). Cell experiments were repeated three times.

We overexpressed KDM6A in PC12 cells overexpressing miR-145 through adenovirus. First, the transfection efficiency (Figure 4(a)) was verified by qRT-PCR and Western blot assay. CCK-8 assay displayed that overexpression of miR-145 elevated the cell viability, and overexpression of KDM6A diminished the viability of cells in the presence of miR-145 overexpression (Figure 4(b)). Flow cytometric data found that overexpression of miR-145 lowered the cell apoptosis, and overexpression of KDM6A could augment apoptosis in the presence of miR-145 overexpression (Figure 4(c)). Consistent results were identified in Western blot assay (Figure 4(d)), where miR-145 could increase Bcl-2 protein expression and decrease Bax and caspase-3, while KDM6A overexpression rescued the changes in the presence of miR-145 overexpression. Moreover, ELISA for cell culture fluid determined that overexpression of miR-145 diminished the expression of proinflammatory cytokines, and overexpression of KDM6A restored the reduction in the presence of miR-145 overexpression (Figure 4(e)). In conclusion, miR-145 protects PC12 cells from LPS-induced cell damage through KDM6A.

### 3.5. miR-145 Downregulates Abcb1a by Inhibiting NOTCH2 Expression through KDM6A

**Figure 5:**
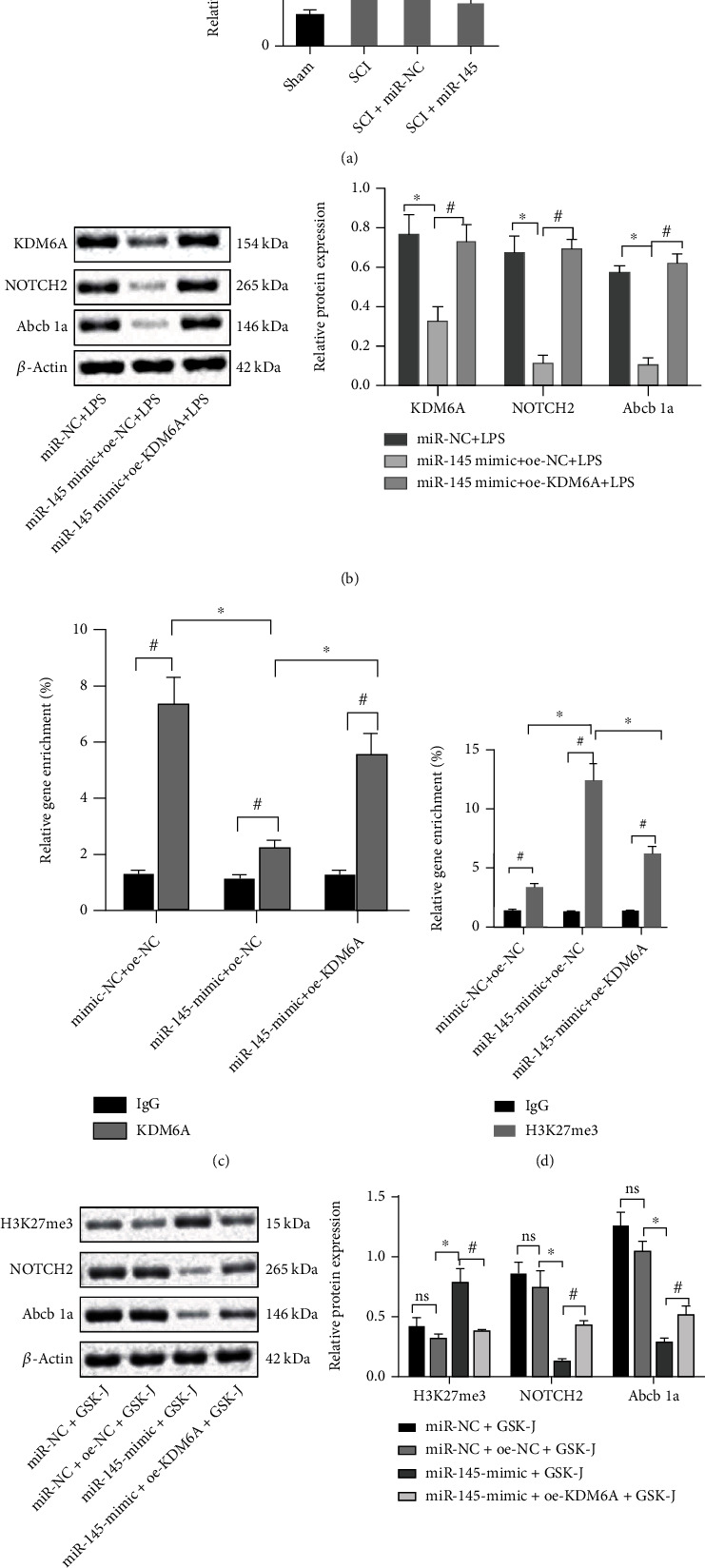
miR-145 downregulates Abcb1a by inhibiting NOTCH2 expression through KDM6A. (a) qRT-PCR to measure the relative expression of KDM6A mRNA in the spinal cord tissue of each group of rats (*n* = 8). (b) Western blot assay to measure the expression of KDM6A, NOTCH2, and Abcb1a proteins in LPS-induced cells. (c) ChIP assay with KDM6A antibody. (d) ChIP assay with H3K27me3 antibody. (e) Western blot assay to measure the protein levels of H3K27me3, NOTCH2, and Abcb1a after treatment with GSK-J. Data were expressed as mean ± standard deviation. Independent sample *t*-test was employed for data comparison between two groups. Data comparison among multiple groups was performed using one-way ANOVA with Tukey's post hoc test. ^∗^*p* < 0.05 versus the sham-operated rats, mimic-NC+oe-NC group, miR-145-mimic+oe-NC group, or mimic-NC+LPS group. ^#^*p* < 0.05 versus the SCI+miR-145 group, mimic-NC+oe-NC+LPS group, miR-145-mimic+LPS group, or IgG group (*F* = 2.25, df = 3, *p* = 0.043). Cell experiments were repeated three times.

We moved to explain the underlying mechanism behind miR-145 regulation. The expression of NOTCH2 in the rat models of SCI was higher than that in the sham-operated rats, and the expression of NOTCH2 in the spinal cord of the model rats overexpressing miR-145 was lower than that in the SCI rats with miR-145 NC (Figure 5(a)). We determined the expression level of NOTCH2 in PC12 cells overexpressing both miR-145 and KDM6A and found that NOTCH2 was diminished by the overexpression of miR-145, and overexpression of KDM6A in combination with overexpression of miR-145 would increase its expression (Figure 5(b)). Therefore, we speculated that miR-145 altered the protein level of NOTCH2 by reducing KDM6A expression and methylation of NOTCH2. We designed ChIP primers at the NOTCH2 promoter site, and the qRT-PCR results showed that, versus IgG antibody, the NOTCH2 gene promoter bound by KDM6A antibody increased, indicating that histone demethylase KDM6A can bind to the NOTCH2 gene promoter region. Overexpression of miR-145 diminished KDM6A expression, and KDM6A binding to the NOTCH2 gene promoter was also reduced. The combination of KDM6A and miR-145 overexpression would augment the binding of KDM6A to the NOTCH2 gene promoter (Figure 5(c)). Detection of H3K27me3 levels in the NOTCH2 gene promoter site revealed that miR-145 overexpression elevated H3K27me3 levels in the NOTCH2 gene promoter site. KDM6A overexpression in combination with overexpression of miR-145 lowered H3K27me3 levels (Figure 5(d)). These results demonstrate that miR-145 affects H3K27me3 levels at the promoter site of the NOTCH2 gene through KDM6A. Cells were treated with histone methylation inhibitor GSK-J, and the expression of NOTCH2 and Abcb1a proteins was determined by Western blot assay. It was found that the addition of H3K27 methylation inhibitors in cells was not significantly diminished before miR-145 was overexpressed, and the change of NOTCH2 was not significant. After overexpression of miR-145, intracellular H3K27me3 levels was elevated, and NOTCH2 expression was downregulated. The addition of inhibitor GSK-J could effectively inhibit intracellular H3K27me3 levels, elevating NOTCH2 expression to a certain extent, and the expression of Abcb1a downstream of NOTCH2 also changed accordingly (Figure 5(e)). Thus, it is concluded that miR-145 downregulates Abcb1a by inhibiting NOTCH2 expression through KDM6A.

### 3.6. miR-145 Attenuates NOTCH2 Expression through KDM6A to Protect PC12 Cells from LPS-Induced Cell Damage

**Figure 6:**
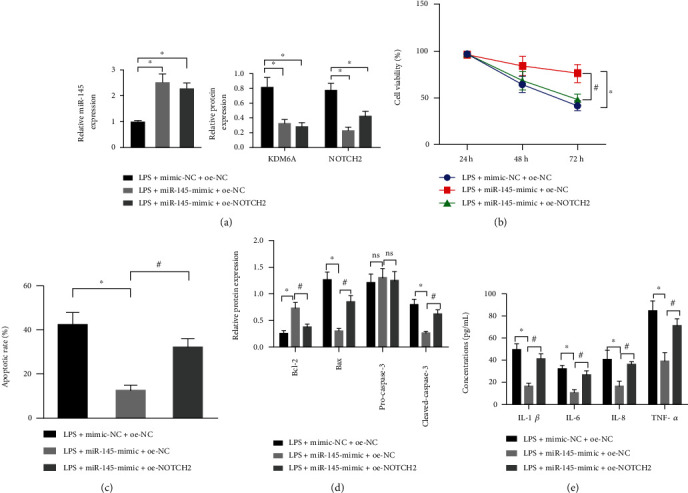
miR-145 attenuates NOTCH2 expression through KDM6A to protect PC12 cells from LPS-induced cell damage. (a) qRT-PCR to measure miR-145 expression (top) and Western blot assay to measure KDM6A and NOTCH2 protein expression (bottom) in LPS-induced cells in response to miR-145 mimic and NOTCH2 overexpression. (b) CCK-8 assay to measure cell viability in LPS-induced cells in response to miR-145 mimic and NOTCH2 overexpression. (c) Flow cytometry to measure cell apoptosis in LPS-induced cells in response to miR-145 mimic and NOTCH2 overexpression. (d) Western blot assay to determine protein levels of apoptosis-related factors in LPS-induced cells in response to miR-145 mimic and NOTCH2 overexpression. (e) ELISA to determine concentration of proinflammatory cytokine release in LPS-induced cell culture fluid in response to miR-145 mimic and NOTCH2 overexpression. Data were expressed as mean ± standard deviation. Independent sample *t*-test was employed for data comparison between two groups. Data comparison among multiple groups was performed using one-way ANOVA with Tukey's post hoc test. Statistical analysis in relation to time-based measurements within each group was realized using repeated measures ANOVA, followed by Bonferroni's post hoc test. ^∗^*p* < 0.05 versus the LPS+mimic-NC+oe-NC group, ^#^*p* < 0.05 versus the LPS+miR-145-mimic+oe-NC group (*F* = 3.15, df = 2, *p* = 0.033). Cell experiments were repeated three times.

We overexpressed miR-145 and NOTCH2 simultaneously in LPS-induced PC12 cells. The qRT-PCR was employed to measure miR-145 expression, and Western blot assay was employed to measure KDM6A and NOTCH2 protein levels to determine transfection efficiency (Figure 6(a)). CCK-8 assay displayed that overexpression of miR-145 elevated the cell viability, and overexpression of both miR-145 and NOTCH2 lowered the cell viability (Figure 6(b)). Flow cytometric data found that overexpression of miR-145 lowered the cell apoptosis and that overexpression of miR-145 and NOTCH2 simultaneously enhanced cell apoptosis (Figure 6(c)). Consistent results were obtained by Western blot assay, where miR-145 could increase Bcl-2 protein expression and decrease Bax and caspase-3. After miR-145 and NOTCH2 were simultaneously overexpressed, the changes were opposite (Figure 6(d)). The concentration of proinflammatory cytokines in cell culture fluid was measured by ELISA, where overexpression of miR-145 diminished the secretion of proinflammatory cytokines, and overexpression of both miR-145 and NOTCH2 made the secretion rise again (Figure 6(e)). The above results indicate that miR-145 attenuates NOTCH2 expression through KDM6A to protect PC12 cells from LPS-induced cell damage.

### 3.7. miR-145 Attenuates NOTCH2 Expression through KDM6A to Augment Neuroprotection in Rats with SCI In Vivo

**Figure 7:**
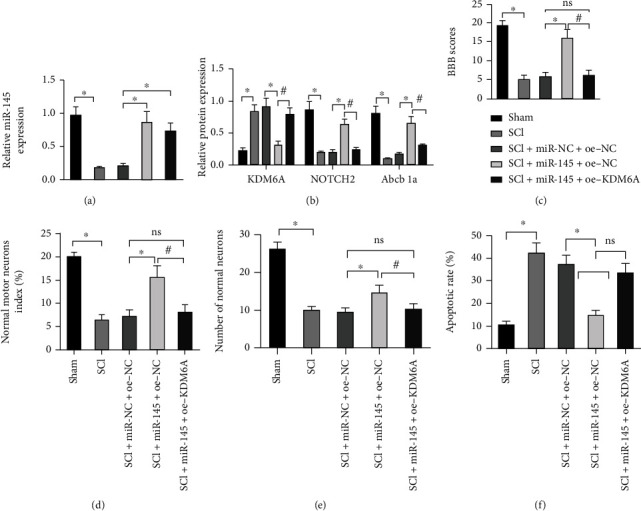
miR-145 confers neuroprotection in rats with SCI by regulating KDM6A *in vivo*. (a) qRT-PCR detection of the relative expression of miR-145 in the spinal cord tissue of each group of rats. (b) Western blot assay to detect the protein expression of KDM6A, NOTCH2, and Abcb1a in the spinal cord tissue of each group of rats. (c) BBB score to evaluate the neurological function of rats of each group. (d) Quantitative analysis for the normal motor neuron index by HE staining in all motor neurons (normal+abnormal neurons). (e) Quantitative analysis for number of normal motor neurons in spinal cord by Nissl staining. (f) Quantitative analysis for the apoptotic rate of neurons of spinal cord of rats by TUNEL assay. Data were expressed as mean ± standard deviation. Data comparison among multiple groups was performed using one-way ANOVA with Tukey's post hoc test. ^∗^*p* < 0.05 versus the sham-operated rats or SCI+miR-NC+oe-NC group, ^#^*p* < 0.05 versus the SCI+miR-145+oe-NC group (*F* = 2.05, df = 1, *p* = 0.032), *n* = 8.

Following the aforementioned *in vitro* experiments, we then validated the miR-145/KDM6A/NOTCH2 axis *in vivo*. After overexpressing miR-145 and KDM6A in SCI rats, we found that miR-145 expression in SCI rats was augmented in the presence of miR-145 overexpression alone or its combination with KDM6A overexpression (*p* < 0.05) (Figure 7(a)). Western blot assay displayed that miR-145 overexpression resulted in downregulated KDM6A and upregulated NOTCH2 and Abcb1a, whereas its combination with KDM6A overexpression led to elevated KDM6A expression and lowered NOTCH2 and Abcb1a expression (Figure 7(b)). Seven days after the operation, the neurological function was assessed using the BBB score (Figure 7(c)). The sham-operated rats had no motor and sensitive dysfunction. The untreated SCI rats score was the same as that of SCI rats of the miR-NC+oe-NC group. Compared to the SCI+miR-145+oe-NC group, the treatment of miR-145+oe-KDM6A diminished BBB score.

Next, we identified through HE staining that the index of normal motor neurons in miR-145-overexpressing SCI rats was higher than that in the untreated SCI rats, whereas the combination of miR-145 and KDM6A overexpression could reverse the increase in normal motor neuron index caused by miR-145 overexpression alone (Figure 7(d)). The results of Nissl staining and normal motor neuron index (Figure 7(e)) were consistent with HE staining results. TUNEL staining results showed that TUNEL-positive cells were brown in the untreated SCI rats and SCI+miR-NC+oe-NC group. However, TUNEL-positive cells were diminished in the SCI+miR-145+oe-NC group. Statistical analysis showed that the number of apoptotic cells in the miR-145+oe-NC group was lower than that in the untreated SCI rats and miR-NC+oe-NC group, and the number of apoptotic cells in the SCI+miR-145+oe-KDM6A group was higher than the SCI+miR-145+oe-NC group (Figure 7(f)). In summary, miR-145 attenuates NOTCH2 expression through KDM6A to augment neuroprotection in rats with SCI *in vivo.*

## 4. Discussion

SCI is commonly caused by an insult inflicted on the spinal cord, leading to dysfunction in neural regeneration and repair [20]. It has been established that neural repair could be augmented through molecular regenerative mechanisms and detrimental events could be prevented through ablating pertinent factors in animal models [21]. Evidence exists proving that miRNA-mRNA interaction has a critical role to play in orchestrating neuron viability and inflammation after SCI, which may potentially be used as therapeutic biomarkers [22, 23]. In this current study, we illuminated the underlying molecular neuroprotective mechanism by which the miR-145/KDM6A/NOTCH2/Abcb1a axis restored neurological functional recovery *in vivo* and alleviated LPS-induced damage in PC12 cells *in vitro*.

In the present study, we developed a rat SCI model by weight drop and an LPS-induced PC12 cell inflammatory injury model. Notably, neuroglial and vascular endothelial cells are stimulated in SCI, releasing inflammatory cytokines and chemokines to induce inflammation; PC12 cell is a neural cell line commonly used to establish *in vitro* LPS-induced neuronal cell inflammation model, in which the expression of inflammatory factors (IL-6, IL-1*β*, IL-8, and TNF-a) are upregulated, consistent with that in SCI animal models [24]. Although whether the two are completely consistent remains uncertain, LPS-treated PC12 cells could be used to mimic the inflammatory response in SCI animals and has thus been used in a number of studies on SCI [25–27].

Accumulating evidence has correlated the epigenetic aberrations of miR-145with neural repair post-SCI. For instance, Geniposide, which upregulates the expression of miR-145, has been reported for alleviating SCI by inhibiting inflammation [12]. It has also been revealed that overexpression of miR-145 reduces the density of astrocytes at the edge of the injured spinal cord and represses the cell proliferation and migration, thereby alleviating SCI [28]. Besides, SCI can cause muscle atrophy, and downregulation of miR-145 has been found in skeletal muscle tissue after SCI, which is closely related to post-SCI pathology of skeletal muscle through targeting Cited2 [29]. In agreement with the evidences, our data demonstrated that miR-145 was poorly expressed in the spinal cord tissue of the SCI rat model and LPS-induced PC12 cells. Besides, upregulation of miR-145 resulted in improved neuron morphology and neurological function in SCI rats, corresponding to elevated cell viability, and reduced apoptosis and inflammation, thereby protecting PC12 cells from LPS-induced cell damage. These results were consistent with the roles of miR-145 involved in the functional recovery of the motor capacity in a rat model of chronic constriction injury, where miR-145 expressed at low levels in the spinal cord tissue of rats [30]. An *in vitro* study further corroborates our findings and reveals the neuroprotective effects of miR-145 on preventing LPS-induced injury in PC12 cells through inactivating the NF-*κ*B and JNK pathways [31]. Therefore, our data strongly support the hypothesis that miR-145 may be involved in the neural repair following SCI. In addition, miR-145 has been reported for reducing neuropathic pain through modulating the AKT3/mTOR or AKT/NF-*κ*B signaling pathway to reduce inflammation and ion channel overexpression [32]. Moreover, miR-145 may also participate in neuronal regeneration after nerve injury, highlighted for its downregulated expression after unilateral sciatic nerve transection [33]. Next, we proceeded to explain the underlying mechanism and found that miR-145 targeted and inhibited KDM6A expression, thus protecting PC12 cells from LPS-induced cell damage. KDM6A is a histone demethylase on the X chromosome, and ablation of KDM6A has been found to repress neuropathology in autoimmune disease *via* inactivating the neuroinflammation signaling pathways [34]. Additionally, as a histone H3K27 demethylase, KDM6A is frequently highly expressed in endothelial cells following SCI, and ablation of KDM6A potentiates the functional recovery following SCI through an interaction with miR-24 [13]. The observations of this study manifested that KDM6A was highly expressed in spinal cord tissues of the SCI rat model and LPS-induced PC12 cells, as reflected by enhanced cell apoptosis and inflammation in the presence of KDM6A overexpression. It has been elucidated that KDM6A enhanced gene transcription through H3K27me3 and intensified IL-6 release through demethylating H3K27me3 at promoter [35]. In relation to this, we also found the promotion of KDM6A in inflammation of LPS-induced PC12 cells, as evidenced by augmented production of proinflammatory cytokines (IL-1*β*, IL-6, IL-8, and TNF-*α*).

Further, our data unraveled that miR-145 downregulates Abcb1a by inhibiting NOTCH2 expression through KDM6A. In agreement with our finding, a previously conducted study by Ikemura et al. has suggested that miR-145 posttranscriptionally mediated the expression and function of Abcb1a [36]. Inflammation triggered by SCI stimulates the expression of Abcb1a, and downregulation of which is a potential strategy to intensify therapeutic drug delivery to the injured spinal cord [16]. Notably, Abcb1a shares a positive correlation with NOTCH2 expression [37]. Suppressed NOTCH signaling activation leads to neural progenitor cell differentiation, but activated NOTCH activities accelerate apoptotic capacities of motor neurons in the ventral spinal cord [38]. Moreover, KDM6A has been reported to elevate NOTCH2 expression through its demethylase function [14]. Accordingly, it is reasonable to state that miR-145 attenuated NOTCH2 expression through KDM6A to protect PC12 cells from LPS-induced cell damage. We also substantiated that miR-145 conferred neuroprotection in rats with SCI by regulating KDM6A-mediated NOTCH2/Abcb1a *in vivo*.

**Figure 8:**
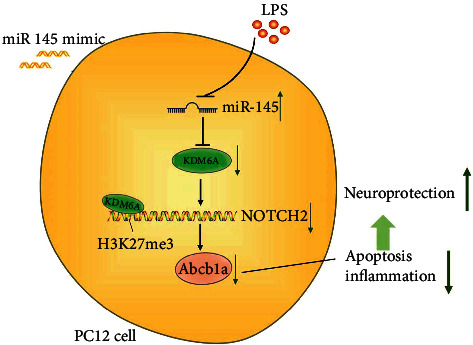
The neuroprotection effects of microRNA-145 in spinal cord injury by downregulating the expression of demethylase KDM6A.

In our current work, we validated the neuroprotective function of miR-145 in reinforcing the neurological functional recovery post-SCI and suppressing LPS-induced damage of PC12 cells through a series of functional experiments both *in vitro* and *in vivo*. Mechanistically, we show that miR-145 targeted and downregulated the demethylase KDM6A expression, thus diminishing the expression of Abcb1a by promoting the methylation of NOTCH2. Through elucidating the regulatory mechanisms of miR-145 in SCI, the study deepened our understanding of the pathogenesis of SCI and provided potential therapeutic targets for SCI treatment (Figure 8). Nonetheless, further experiments are required for detection of the localization of miR-145 by in situ hybridization, so as to identify the aberrant expression of miR-145 in specific types of cells in spinal cord tissues. Besides, more animal experiments as well as long-term clinical trials are needed to validate whether miR-145 and related biomarkers could be clinically applied for SCI. In addition, this study confirmed that miR-145 can promote neuroprotection in rats with spinal cord injury, while whether the therapeutic target is applicable to human beings requires to be further verified.

## Figures and Tables

**Table 1:** Primer sequences for RT-qPCR.

Gene	Primer sequence	
miR-145	Forward	5′- ATCAAGATCAGAGACTCTGCTC-3′
	Reverse	5′- GTGCAGCCGTAGGCTCCGCTC-3′

KDM6A	Forward	5′-CTGAGTCTGACAAAGCCTTC-3′
	Reverse	5′- CTGGTCTGATAGCTCGTCAC-3′

IL-1*β*	Forward	5′-CATGATCCGAGATGTGGAACYGGC -3′
	Reverse	5′-CTGGCTCAGCCACTCCAGC-3′

IL-6	Forward	5′-ACTCACCTCTTCAGGTTG-3′
	Reverse	5′-CCATCTTTGGAAGGTTCAGGTTG -3′

IL-8	Forward	5′- TTTTGCCAAGGAGTGCTAAAGA -3′
	Reverse	5′- AACCCTCTGCACCCAGTTTTC-3′

TNF-*α*	Forward	5′- CATGATCCGAGATGTGGAACTGGC-3′
	Forward	5′-CTGGCTCAGCCACTCCAGC-3′

U6	Forward	5′- GTGCAGGGTCCGAGGT-3′
	Reverse	5′- CTCGCTTCGGCAGCACA-3′

GAPDH	Forward	5′- GGAGCGAGATCCCTCCAAAAT-3′
	Reverse	5′-GGCTGTTGTCATACTTCTGG-3′

## Data Availability

The datasets generated/analyzed during the current study are available.
